# Supplementary material for: Leaf damage is associated with alteration of microbiome composition and abundance in the Spartina alterniflora phyllosphere
Source: Microbiol Spectr. 2026 Oct 9;14(11):e01597-26. doi: 10.1128/spectrum.01597-26 (PMC13729216; doi:10.1128/spectrum.01597-26)
Supplement: Supplementary file 1 [file reviewer-comments.pdf]

ASM | Microbiology Spectrum

Leaf Damage Is Associated with Alteration of Microbiome Composition and Abundance in the *Spartina alterniflora* Phyllosphere

Jose Rolando, Anna Carnes, Michael Hodges, Heather Joesting, and Joel Kostka

Corresponding Author(s): Jose Rolando, University of Florida Department of Microbiology and Cell Science

Review Timeline:

Submission Date:	May 11, 2026
Editorial Decision:	July 7, 2026
Revision Received:	August 15, 2026
Accepted:	August 18, 2026

Editor: Frédérique Reverchon

Reviewer(s): Disclosure of reviewer identity is with reference to reviewer comments included in decision letter(s). The following individuals involved in review of your submission have agreed to reveal their identity: Elizabeth Hernández Alarcón (Reviewer #1)

Transaction Report:

(Note: With the exception of the correction of typographical or spelling errors that could be a source of ambiguity, letters and reports are not edited. The original formatting of letters and referee reports may not be reflected in this compilation.)

DOI: https://doi.org/10.1128/spectrum.01597-26

Re: Spectrum01597-26 (Leaf Damage Is Associated with Microbiome Dysbiosis in the *Spartina alterniflora* Phyllosphere)

Dear Dr. Jose Rolando:

Thank you for the privilege of reviewing your work. Below you will find my comments, instructions from the Spectrum editorial office, and the reviewer comments.

I have received comments from two independent reviewers.
They both highlight the merits of your work but point out the need to emphasise the relevance of your study in the context of ecosystem restoration and contribution to the field.

Please return the manuscript within 60 days; if you cannot complete the modification within this time period, please contact me. If you do not wish to modify the manuscript and prefer to submit it to another journal, notify me immediately so that the manuscript may be formally withdrawn from consideration by Spectrum.

Revision Guidelines
To submit your modified manuscript, log into the submission site at https://spectrum.msubmit.net/cgi-bin/main.plex. Go to Author Tasks and click the appropriate manuscript title to begin. The information you entered when you first submitted the paper will be displayed; update this as necessary. Note the following requirements:

• Upload point-by-point responses to the issues raised by the reviewers in a file named "Response to Reviewers," NOT in your cover letter.
• Upload a compare copy of the manuscript (without figures) as a "Marked-Up Manuscript" file.
• Upload a clean .DOC/.DOCX version of the revised manuscript and remove the previous version.
• Each figure must be uploaded as a separate, editable, high-resolution file (TIFF or EPS preferred), and any multipanel figures must be assembled into one file.
• Any supplemental material intended for posting by ASM should be uploaded with their legends separate from the main manuscript. You can combine all supplemental material into one file (preferred) or split it into a maximum of 10 files with all associated legends included.

For complete guidelines on revision requirements, see our Submission and Review Process webpage. Submission of a paper that does not conform to guidelines may delay acceptance of your manuscript.

Data availability: ASM policy requires that data be available to the public upon online posting of the article, so please verify all links to sequence records, if present, and make sure that each number retrieves the full record of the data. If a new accession number is not linked or a link is broken, provide Spectrum production staff with the correct URL for the record. If the accession numbers for new data are not publicly accessible before the expected online posting of the article, publication may be delayed; please contact production staff (Spectrum@asmusa.org) immediately with the expected release date.

Publication Fees: For information on publication fees and which article types are subject to charges, visit ourwebsite. If your manuscript is accepted for publication and any fees apply, you will be contacted separately about payment during the production process; please follow the instructions in that e-mail. Arrangements for payment must be made before your article is published.

ASM Membership: Corresponding authors may join or renew ASM membership to obtain discounts on publication fees. Need to upgrade your membership level? Please contact Customer Service at Service@asmusa.org.

The ASM Journals program strives for constant improvement in our submission and publication process. Please tell us how we can improve your experience by taking this quick Author Survey.

Thank you for submitting your paper to Spectrum.

Sincerely,
Frédérique Reverchon
Editor
Microbiology Spectrum

Reviewer #1 (Comments for the Author):

The study investigated the microbiome of bacteria and fungi in the phyllosphere of Spartina alternifolia under different

phenotypes, stress conditions, and origins. They find that plant origin (i.e., greenhouse vs. natural marsh), plant health status (i.e., healthy vs. damaged), and plant phenotype (i.e., short vs. tall Spartina) affect microbial relative abundance, alpha diversity, and community composition in the S. alterniflora phyllosphere. The study concludes that the damage caused to the leaves is due to dysbiosisAlthough the manuscript is well written, as a reader, I have doubts about the relevance of the study; they argue that the knowledge generated could be used to recommend restoration practices for salt marshes. However, although in the discussion they address the possibility of inoculating greenhouse plants to make them more resistant, I do not believe that the knowledge generated in the work help to reach this goal, because they talk about beneficial microorganisms and this study only generate diversity knowledge not functional diversity.

The title should be less conclusive and reflect more the results of the study, which is actually a study of the microbial microbiome in the phyllosphere of Spartina alternifolia from different origins, phenotypes, and health states. The conclusions are a bit obvious, coinciding with what has been found in previous studies with different plants species, which casts doubt on the rationale of the work. I believe that the relevance and contribution of the work should be better addressed.

Reviewer #2 (Comments for the Author):

1. Please clarify why there were not short and tall phenotype in greenhouse?
2.There are too many references. It is strongly recommended to reduce to less than 50, and the authors are strongly encouraged to incorporate the latest research progress from 2021-2026 to ensure the context and discussion are current.
3.The authors are recommended to perform Redundancy Analysis (RDA) or Canonical Correspondence Analysis (CCA) to identify the key environmental factors shaping prokaryotic and fungal phyllosphere community composition in natural S. alterniflora plants.
4. Line 269-270: "where periwinkle snail density is highest" should be revised to "where periwinkle snail density is the highest" .

This study investigates the response of the native leaf-associated microbiome to leaf damage resulting from disease and environmental stress. The authors evaluated the changes in prokaryotic and fungal relative abundance, diversity, and community composition in the *Spartina alterniflora* phyllosphere microbiome. The topic is relevant and meaningful, given the ecosystem services of *S. alterniflora* to global and local communities. Furthermore, the research topic is well-aligned with the scope of Microbiology Spectrum. The core scientific findings appear promising, the manuscript is well-structured and logically organized. However, some minor issues need to be addressed before further consideration. Specific comments are provided below.

1. Please clarify why there were not short and tall phenotype in greenhouse?

2.There are too many references. It is strongly recommended to reduce to less than 50, and the authors are strongly encouraged to incorporate the latest research progress from 2021-2026 to ensure the context and discussion are current.

3.The authors are recommended to perform Redundancy Analysis (RDA) or Canonical Correspondence Analysis (CCA) to identify the key environmental factors shaping prokaryotic and fungal phyllosphere community composition in natural *S. alterniflora* plants.

4. Line 269-270: “where periwinkle snail density is highest” should be revised to “where periwinkle snail density is the highest” .

RESPONSE TO REVIEWERS

Spectrum01597-26 (Leaf Damage Is Associated with Microbiome Dysbiosis in the *Spartina alterniflora* Phyllosphere)

Editor comment:
Dear Dr. Jose Rolando:

Thank you for the privilege of reviewing your work. Below you will find my comments, instructions from the Spectrum editorial office, and the reviewer comments.

I have received comments from two independent reviewers.
They both highlight the merits of your work but point out the need to emphasise the relevance of your study in the context of ecosystem restoration and contribution to the field.

Please return the manuscript within 60 days; if you cannot complete the modification within this time period, please contact me. If you do not wish to modify the manuscript and prefer to submit it to another journal, notify me immediately so that the manuscript may be formally withdrawn from consideration by Spectrum.

Reply: We appreciate the positive assessment of our work. We have improved our manuscript based on the comments made by the reviewers, especially by emphasizing the novelty and relevance of our work on saltmarsh restoration (e.g., lines 62-65, 238-239, 362-365 in the marked manuscript). Please find detailed responses to each of their comments below.

Reviewer #1 (Comments for the Author):

Reviewer 1, Comment 1:

The study investigated the microbiome of bacteria and fungi in the phyllosphere of Spartina alternifolia under different phenotypes, stress conditions, and origins. They find that plant origin (i.e., greenhouse vs. natural marsh), plant health status (i.e., healthy vs. damaged), and plant phenotype (i.e., short vs. tall Spartina) affect microbial relative abundance, alpha diversity, and community composition in the S. alterniflora phyllosphere. The study concludes that the damage caused to the leaves is due to dysbiosis.

Reply: Thank you for the summary of our work. However, we may not have stated this clearly enough in our manuscript. We do not claim that leaf damage was caused by microbiome dysbiosis. We assumed that leaf damage was caused by previously described processes in salt marshes, such as primary fungal infection, abiotic environmental stressors (salinity, sulfide), or herbivory by insects and/or snails. To make this clearer, and avoid misinterpretation of our

results, we have removed the concept of dysbiosis from the title, importance and discussions section. Please see lines 276-281: "*Increased alpha diversity following leaf damage is more consistent with niche expansion than with pathogen bloom, suggesting that leaf damage in S. alterniflora may not follow the same ecological trajectory as dysbiosis described in other systems. Thus, although we observed a profound change in the composition and abundance of microbes following plant damage, we could not confirm further health deterioration of the plant host by microbiome dysbiosis***" and new title: "***Leaf Damage Is Associated with Alteration of Microbiome Composition and Abundance in the Spartina alterniflora Phyllosphere***".**

Reviewer 1, Comment 2:

Although the manuscript is well written, as a reader, I have doubts about the relevance of the study; they argue that the knowledge generated could be used to recommend restoration practices for salt marshes. However, although in the discussion they address the possibility of inoculating greenhouse plants to make them more resistant, I do not believe that the knowledge generated in the work help to reach this goal, because they talk about beneficial microorganisms and this study only generate diversity knowledge not functional diversity.

Reply: The novelty of this study lies in the fact that it is arguably the first study investigating the environmental factors that mediate microbiome assembly of *Spartina alterniflora* in the wild and under greenhouse conditions, specifically using cultivation-independent techniques. It is true that we have not directly investigated the use of beneficial microbes for restoration activities, since this was outside the scope of the study. However, this study provides a baseline of diversity and abundance patterns across leaf health status, plant phenotype, and wild vs. greenhouse plants, which will inform the development of future microbiome-based applications for this plant. We have strengthened the text to better highlight the novelty of our work on lines 238-239, as well as the need for further research on the use of biological applications for *S. alterniflora* restoration in lines 353-356.

Reviewer 1, Comment 3:

The title should be less conclusive and reflect more the results of the study, which is actually a study of the microbial microbiome in the phyllosphere of Spartina alternifolia from different origins, phenotypes, and health states.

Reply: We have modified the title as suggested by the reviewer. The new title removes the concept of dysbiosis: *"Leaf Damage Is Associated with Alteration of Microbiome Composition and Abundance in the Spartina alterniflora Phyllosphere".*

Reviewer 1, Comment 4:

The conclusions are a bit obvious, coinciding with what has been found in previous studies with different plants species, which casts doubt on the rationale of the work. I believe that the relevance and contribution of the work should be better addressed.

Reply: Please refer to Comment 2 as a response to the novelty and contribution of this study.

Reviewer #2 (Comments for the Author):

Reviewer 2, Comment 1:

1. Please clarify why there were not short and tall phenotype in greenhouse?

Reply: The short and tall phenotypes of *Spartina alterniflora* are phenotypic expressions of this plant under contrasting environmental conditions experienced in the salt marsh. Because greenhouse-grown *Spartina* for restoration activities does not mimic the environmental conditions experienced by the plant in situ (i.e., tidal flushing, sediment anoxia, biotic interactions with invertebrates and microbes, etc.), we did not apply the short or tall phenotypic classification to greenhouse plants; instead, we state that greenhouse plants represent a distinct phenotypic expression of the plant. This has been added to the text in lines 182-186.

Reviewer 2, Comment 2:

2.There are too many references. It is strongly recommended to reduce to less than 50, and the authors are strongly encouraged to incorporate the latest research progress from 2021-2026 to ensure the context and discussion are current.

Reply: We thank the reviewer for this comment. Microbiology Spectrum Research Articles do not have a specified reference limit. Given the interdisciplinary scope of our study integrating salt marsh microbial, plant, and ecosystems/restoration ecology, we believe the current reference list is necessary to support our background, methods and discussion. However, we have still revised our reference list and removed 7 references that were redundant, reducing the references from 69 to 62 citations. Also, following the author suggestion to incorporate recent references, we have added references the following reference:

Yang X, Dai Z, Yuan R, Guo Z, Xi H, He Z, Wei M. 2023. Effects of Salinity on Assembly Characteristics and Function of Microbial Communities in the Phyllosphere and Rhizosphere of Salt-Tolerant *Avicennia marina* Mangrove Species. Microbiol Spectr 11:e03000-22.

Unfortunately, as we make the case in this manuscript, there are few studies of the salt marsh phyllosphere microbiome, which prevents us from citing more recent studies.

Reviewer 2, Comment 3:

3.The authors are recommended to perform Redundancy Analysis (RDA) or Canonical Correspondence Analysis (CCA) to identify the key environmental factors shaping prokaryotic and fungal phyllosphere community composition in natural S. alterniflora plants.

Reply: Unfortunately, we do not have corresponding environmental metadata (e.g., sediment carbon, nitrogen, redox, pH, porewater nutrients, etc.) for the sampling events when phyllosphere data were collected (2021–2022). Thus, we could not perform RDA or CCA analyses for this dataset. The same transects at Sapelo and Skidaway Island were sampled in 2018–2019 for sediment and root microbiomes, and differences in environmental conditions between tall and short *Spartina* sites can be found there (Rolando et al., 2022). Although environmental conditions should be similar than 2018-2019, we do not think is appropriate to correlate phyllosphere microbiome data from 2021–2022 with environmental metadata from 2018–2019. PERMANOVA analysis in our study, however, does show that *Spartina* phenotype (which includes the strong environmental conditions that shape it) is one of the most important factors determining phyllosphere microbiome structure for both prokaryotic and fungal communities (Table 2). Please see lines 170-174.

Reviewer 2, Comment 4:

4. Line 269-270: "where periwinkle snail density is highest" should be revised to "where periwinkle snail density is the highest" .

Reply: Thank you for catching the typo. It has been corrected. Please see lines 290-291.

Re: Spectrum01597-26R1 (**Leaf Damage Is Associated with Alteration of Microbiome Composition and Abundance in the *Spartina alterniflora* Phyllosphere**)

Dear Dr. Jose L. Rolando:

Thank you for providing the revised version of your manuscript.
All comments raised by the reviewers have been addressed and I am glad to recommend your manuscript for publication in Microbiology Spectrum.

Your manuscript has been accepted, and I am forwarding it to the ASM production staff for publication. Your paper will first be checked to make sure all elements meet the technical requirements. ASM staff will contact you if anything needs to be revised before copyediting and production can begin. Otherwise, you will be notified when your proofs are ready to be viewed.

Data Availability: ASM policy requires that data be available to the public upon online posting of the article, so please verify all links to sequence records, if present, and make sure that each number retrieves the full record of the data. If a new accession number is not linked or a link is broken, provide production staff with the correct URL for the record. If the accession numbers for new data are not publicly accessible before the expected online posting of the article, publication may be delayed; please contact ASM production staff immediately with the expected release date.

Publication Fees: For information on publication fees and which article types have charges, please visit our website. We have partnered with Copyright Clearance Center (CCC) to collect author charges. If fees apply to your paper, you will receive a message from no-reply@copyright.com with further instructions. For questions related to paying charges through RightsLink, please contact CCC at ASM_Support@copyright.com or toll free at +1-877-622-5543. CCC makes every attempt to respond to all emails within 24 hours.

ASM Membership: Corresponding authors may join or renew ASM membership to obtain discounts on publication fees. Need to upgrade your membership level? Please contact Customer Service at Service@asmusa.org.

Embargo Policy: A press release may be issued as soon as the manuscript is posted on the Spectrum Latest Articles webpage. The corresponding author will receive an email with the subject line "ASM Journals Author Services Notification" when the article is available online.

Cover Image Submissions: If you would like to submit a potential Cover Image, please email a file and a short legend to spectrum@asmusa.org. Please note that we can only consider images that (i) the authors created or own and (ii) have not been previously published. By submitting, you agree that the image can be used under the same terms as the published article. Image File requirements: TIF/EPS, 7.5 inches wide by 8.25 inches tall (at least 2,250 pixels wide by 2,475 pixels tall), minimum 300 dpi resolution (600 dpi preferred), RGB, and no figure elements, e.g., arrows or panel labels. The legend should be a short description of the image, 1-2 sentences recommended. Please use the ASM Journals Cover Image Sizing Tool (https://journals.asm.org/pb-assets/html/asm-cover-sizing-tool.html) to ensure that your proposed cover image meets our size requirements.

The ASM Journals program strives for constant improvement in our submission and publication process. Please tell us how we can improve your experience by taking this quick Author Survey.

Thank you for submitting your paper to Spectrum.

Sincerely,
Frédérique Reverchon
Editor
Microbiology Spectrum
