# Supplementary material for: Leaf damage is associated with alteration of microbiome composition and abundance in the Spartina alterniflora phyllosphere
Source: Microbiol Spectr. 2026 Oct 9;14(11):e01597-26. doi: 10.1128/spectrum.01597-26 (PMC13729216; doi:10.1128/spectrum.01597-26)
Supplement: Supplementary file 2 [file spectrum.01597-26-s0001.pdf]

Supplementary Material for Rolando et al., *Leaf damage is associated with alteration of microbiome composition and abundance in the Spartina alterniflora phyllosphere*

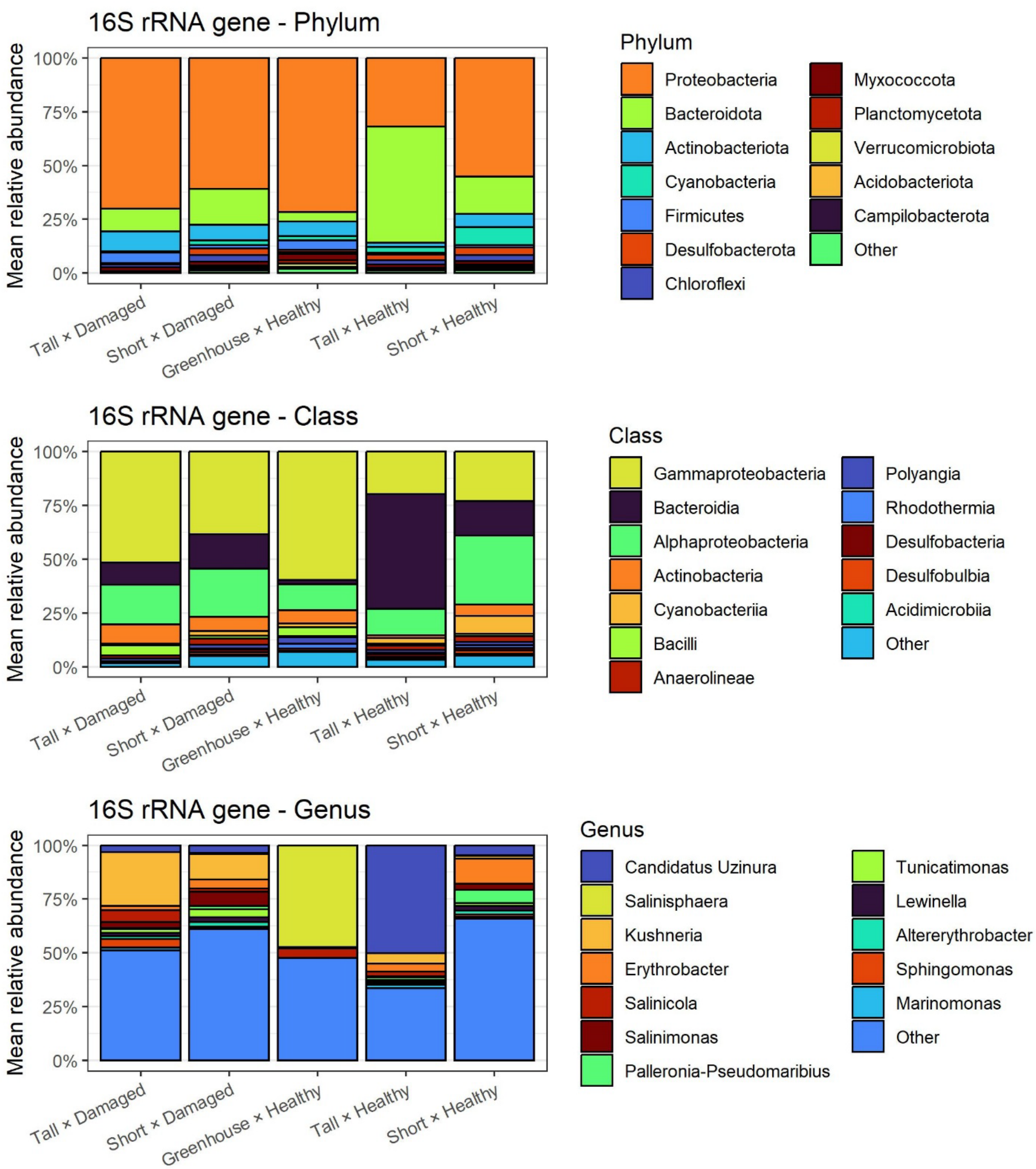

Figure S1. Prokaryotic composition of the *Spartina alterniflora* phyllosphere microbiome

Stacked bar plots representing the mean relative abundance of prokaryotic taxa detected by 16S rRNA gene amplicon sequencing in the phyllosphere of *Spartina alterniflora*. Plant organelle 16S

rRNA gene reads were discarded before plotting. Taxonomic composition is shown at the (A) phylum, (B) class, and (C) genus levels. Samples are grouped by plant phenotype (Tall vs. Short), plant health status (Healthy vs. Damaged), and greenhouse-grown plants where indicated. Only the top 12 most abundant taxa at each rank are shown individually, while the remaining low-abundance taxa are grouped into the “Other” category.

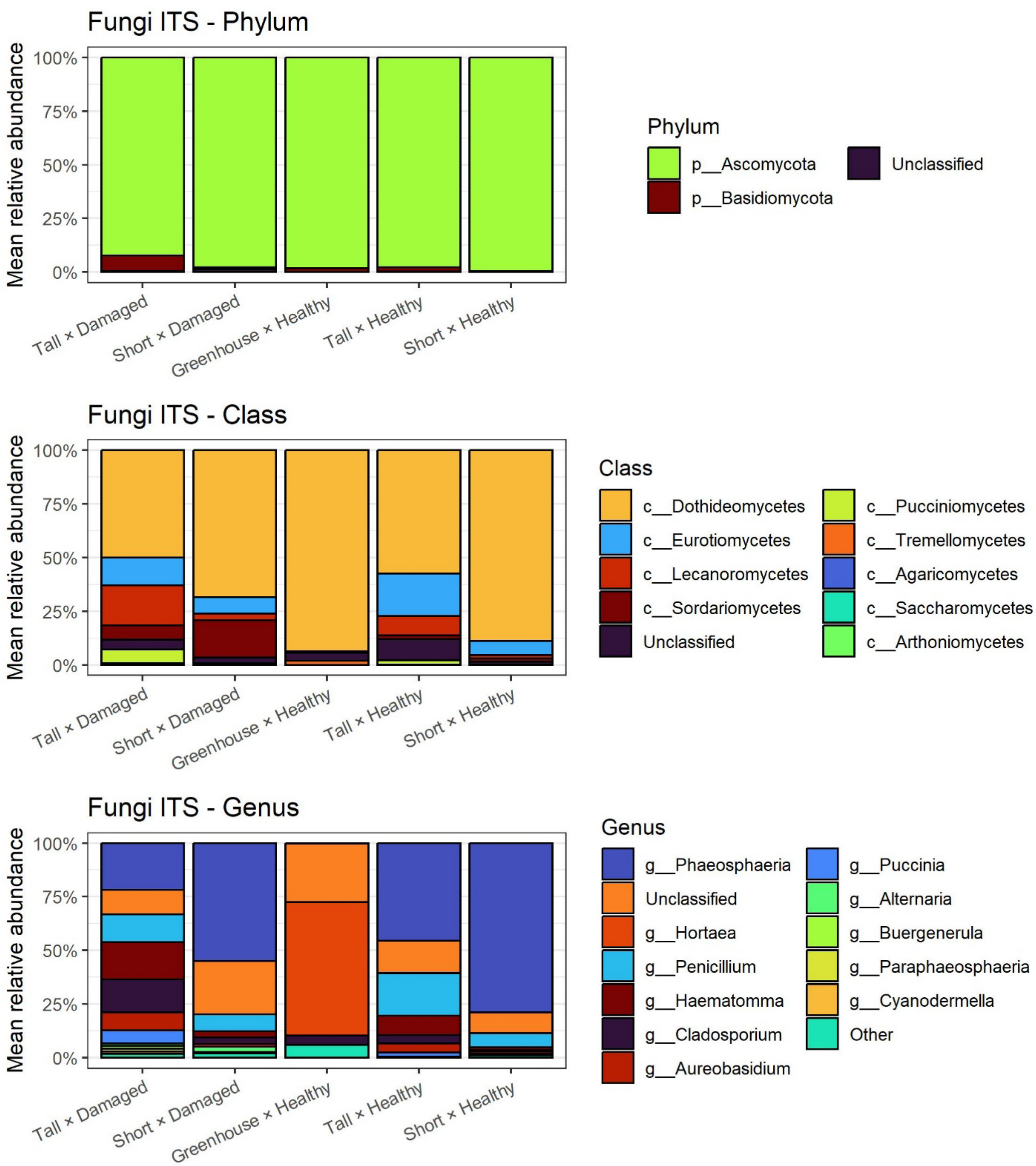

Figure S2. Fungal composition of the *Spartina alterniflora* phyllosphere microbiome

Stacked bar plots representing the mean relative abundance of fungal taxa detected by ITS region amplicon sequencing in the phyllosphere of *Spartina alterniflora*. Plant ITS reads were discarded before plotting. Taxonomic composition is shown at the (A) phylum, (B) class, and (C) genus levels. Samples are grouped by plant phenotype (Tall vs. Short), plant health status (Healthy vs.

Damaged), and greenhouse-grown plants where indicated. Only the top 12 most abundant taxa at each rank are shown individually, while the remaining low-abundance taxa are grouped into the “Other” category.

Table S1: Two-way analysis of variance (ANOVA) of the Shannon diversity index as explained by Spartina phenotype, plant health status, and its interaction

	16S rRNA		ITS	
Factor	F-value	p-value	F-value	p-value
Spartina Phenotype	76.67	2.7E-15	2.17	0.143
Plant Health	5.70	0.018	7.33	0.007
Interaction	9.33	0.003	0.04	0.850
